# Dr. Francis Peabody

**Published:** 1897-11

**Authors:** 


### Obituary.

### REPORT OF THE COMMITTEE ON NECROLOGY OF THE NATIONAL ASSOCIATION OF DENTAL FACULTIES.

### DR. FRANCIS PEABODY.

WHEREAS, Death has taken from among us Dr. Francis Peabody, of Louisville, Ky.; and

WHEREAS, We feel that in his death the profession has sustained the loss of an able practitioner and teacher; therefore be it

*Resolved*, That we tender to his bereaved family our heartfelt sympathy, and that we cause the resolutions to be entered upon the minutes of this Association; and be it further

*Resolved*, That a copy of these resolutions be sent to the dental journals for publication.

